# A compact evolved antenna for 5G communications

**DOI:** 10.1038/s41598-022-14447-9

**Published:** 2022-06-20

**Authors:** I. Marasco, G. Niro, V. M. Mastronardi, F. Rizzi, A. D’Orazio, M. De Vittorio, M. Grande

**Affiliations:** 1 Politecnico di Bari, Via E. Orabona 4, 70125 Bari (BA), Italy; 2 Center for Biomolecular Nanotechnologies, Istituto Italiano di Tecnologia (IIT), Via E. Barsanti 14, 73010 Arnesano (LE), Italy; 3 Dipartimento di Ingegneria Dell’innovazione, Università del Salento, Campus Ecotekne, Via Monteroni, Lecce (LE), Italy

**Keywords:** Electrical and electronic engineering, Design, synthesis and processing

## Abstract

Flexible and bendable electronics are gaining a lot of interest in these last years. In this scenario, compact antennas on flexible substrates represent a strategical technological step to pave the way to a new class of wearable systems. A crucial issue to overcome is represented by the poor radiation properties of compact antennas, especially in the case of flexible and thin substrates. In this paper, we propose an innovative design of a miniaturized evolved patch antenna whose radiation properties have been enhanced with a Split Ring Resonator (SRR) placed between the top and the ground plane. The antenna has been realized on a flexible and biocompatible substrate polyethylene naphthalate (PEN) of 250 μm by means of a new fabrication protocol that involves a three-layer 3D-inkjet printing and an alignment step. The antenna has been characterized in terms of the scattering parameter S_11_ and the radiation pattern showing a good agreement between simulations and measurements.

## Introduction

With the advent of 5G and new 6G network infrastructures, flexible and bendable IoT devices are becoming increasingly popular^1,2^. In this regard, the conception of wearable and bendable antennas is a crucial issue to overcome^3,4^. Scientific efforts concerned the design of miniature flexible antennas to cope with modern requirements such as wearability.

Due to the high demand coming from the market, rapid prototyping and cost-effective technologies for antenna fabrication are becoming crucial and, hence, multiple technologies have to be combined to achieve the very strict goals required by antenna design. From one side, the footprint of the antenna can be reduced increasing the operating frequency^5^. However, the higher is the transmission frequency the more probable is the insurgence of disturbing phenomena such as multipath fading and attenuation^6^. The second alternative is antenna miniaturization. This technique aims to decrease the resonance of antennas by increasing its electrical length, so with the same geometrical dimension antennas can radiate at a lower frequency. The main drawbacks of this strategy are the degrading of the radiation properties and bandwidth reduction. In addition to compact footprints, optimal radiation properties are needed. These requirements increase the difficulty of the design procedure since the geometry of the antenna must be efficiently optimized, typically and especially on very thin substrates.

In this context, artificial intelligence (AI) comes to aid. AI algorithms have been used for years to solve a huge variety of optimization problems, including antennas design. Among other methods, the genetic algorithm (GA) is one of the most used approaches regarding different electromagnetic problems such as photonics^7^ and antennas^8–11^.

The genetic algorithm generates a set of trivial solutions, i.e. antenna geometries, which evolves step by step until the best solution is reached. Antennas generated according to this approach are said to be evolved^12^. Miitra^13^ was one of the first authors to exploit this line of research. Reference^8^ reports a tutorial about GA applied to this kind of applications while reference^9^ details the design of antennas employing GA and the Method of Moments (MoM). In^10^, the GA is applied to miniaturize a patch antenna for cardiac pacemakers operating at 402–405 MHz. The coding scheme used to treat the problem consists of subdividing the patch into smaller sub-patches. Each sub-patch can be either metal or air. The same approach has also been used in^11^, where the genetic algorithm has been applied to a patch antenna shifting its resonance from 4.8 GHz to 2.16 GHz.

It has been demonstrated that the design of antennas by means of genetic algorithms is a good choice; however, only the miniaturization cannot be pushed far without suffering from low radiation efficiencies.

An approach used for improving this feature is the introduction of metamaterials. Each material in nature is composed of atoms and their properties and spatial orientations affect its electrical behavior. The idea behind the metamaterials is the logical expansion of this concept at a macroscopic scale. By creating an array of single cell having specific properties it is possible to generate tailored electrical and magnetic characteristics of the entire structure.

Metamaterials are applied to a huge variety of fields and one of the most promising regards the enhancement of radiation properties of small antennas.

Split Ring Resonators (SRRs) represent an optimal solution since their planar geometry and easy integration simplifies the fabrication process^14^. In particular, SRRs can be used to realized Artificial Magnetic Conductor (AMC) layers having effective permeability near to zero, i.e. Zero Index metamaterials (ZIMs). Recent studies employ ZIMs to improve the radiation properties of antennas by placing such supermaterials between the top layer of the antenna and the ground plane^15–22^. In this case, the ZIM acts as a very precise mirror that does not introduce phase variations in the reflected electromagnetic waves, improving the radiation characteristics of the ground plane on the bottom of the antenna. A problem arising from the use of metamaterials is that the inclusion of a superstrate adds a metallic layer to the stack composing the antenna, hence an alignment process must be performed.

A very attractive technological process for the fabrication of flexible antennas even of multilayers stacks^23,24^, is the ink-jet printing^25^, as it is the most economical, the fastest, and the cleanest solution. By this technique, the antennas are realized by depositing conductive inks through hundreds of nozzles which are mechanically controlled. Despite its optimal characteristics, this method does face some issues: the quality of the prints depends on the viscosity and the size of the droplet of the conductive ink^25^.

In addition, in the case of multilayers antennas fabricated on commercial substrates not direct printable, the alignment step becomes difficult. This happens because it is necessary to perform two different printing processes on both faces of the same dielectric. This last case can be the case of metamaterial antennas designed on stretchable substrates.

In^26^ an example of a flexible antenna enhanced with a metamaterial has been reported. The antenna is composed of a polyimide substrate with a top radiating element placed on a second substrate with a 3 × 3 array of metamaterial structures and a ground plane at the bottom. However, in this case, a precise alignment is not strictly necessary since the superstate needs only the metallization of its bottom surface. Moreover, the two layers are not fully integrated but are just placed on each other.

As a consequence, even though in literature there exist a huge number of antennas realized using GA, metamaterials and inkjet printing, to the best of our knowledge, an antenna that exploits these three technologies jointly has not been reported yet.

The biggest issue to overcome is the lack of the rapid prototyping fabrication techniques on flexible substrates of a process characterized by multilayer printing with an optimal alignment between all the parts composing the antenna. In this work, we propose a flexible multilayer ink-jet printed evolved antenna. The stack is composed of five layers (three metallic and two dielectric). The radiative layer (i.e. top layer) is designed by using a genetic algorithm in order to miniaturize its footprint. The gain is enhanced by a split ring resonator which acts as a metamaterial and, at the bottom, the ground plane is placed to minimize the back radiation. The working frequency is in the sub-6 GHz band of the 5G spectrum, in particular, around 4 GHz.

The metallic layers are fabricated by means of a multi-material 3D printer which requires a precise alignment process on a 250 μm Polyethilene Naphtalate (PEN) substrate. The dielectric layers are attached to each other by a Polydimethylsiloxane (PDMS) adhesive interlayer of 40 μm.

Finally, the antenna has been characterized in terms of the S_11_ parameter and 2D radiation patterns by means of VNA. The paper is organized as follows: in “Genetic algorithm” we described briefly the principal steps of the GA, in “Antenna design and Split ring resonator”, the antenna design is reported, in “Fabrication” the fabrication steps are described, in “Characterization” the characterization of the prototype is detailed and, finally, in “Conclusion” conclusions are presented.

## Genetic algorithm

**Figure 1:**
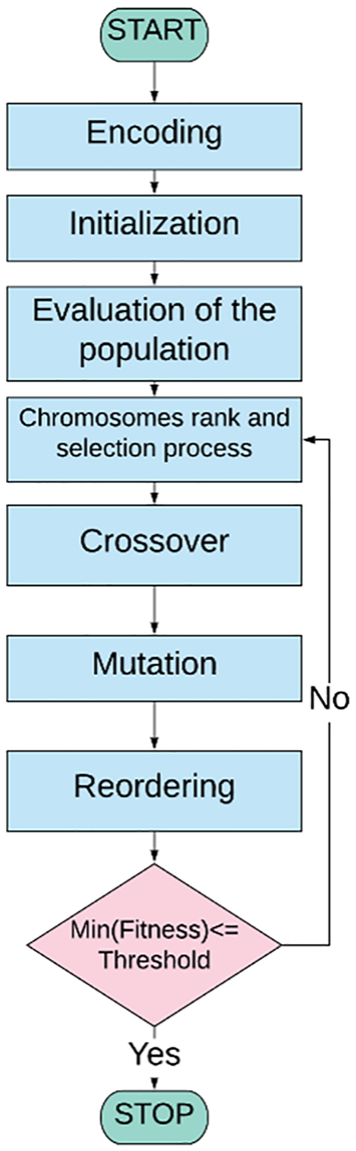
Flowchart of the Genetic Algorithm.

The Genetic Algorithm first formalized in^27^ is a robust global optimization method based on Darwinian laws. The process starts from an initial random binary population (parents) that evolves iteratively towards the optimal solution under the selective influence of a cost function. The genetic algorithm works through eight main steps, as shown in Fig. 1.

(1) *Encoding* the problem is schematized as a function of general parameters. A single parameter is treated as a gene. Generally, binary values are preferable to be used for the genes. Their arrangement (i.e. all the genes) forms the chromosome which describes the partial solution.

(2) *Initialization* the initial population is generated. The set of trivial solution is composed of random chromosomes.

(3) *Evaluation of the population* at this step, the fitness function is applied to all the elements of the population. At the end of this process, a direct correspondence between each chromosome and its relative fitness value is obtained, which quantifies the proximity of the trivial to the real solution of the problem.

(4) *Chromosomes rank and selection process* the chromosomes are grouped two by two. The selection can be performed randomly or by considering their fitness values.

(5) *Crossover* from each couple of chromosomes, the offsprings are generated. The descendants are created by mutual recombination of the genes of the initial chromosomes forming a new set of gene values starting from the parents.

(6) *Mutation* the set of trivial solutions is perturbated by the insertion of random variations which can speed up the convergence of the algorithm. Mutations can be applied to a single gene or multiple chromosomes and are strictly dependent on the nature of the problem. In general, the presence of mutations avoids stalling the solver in local minimum, however, a rate too high can degrade the performance.

(7) *Reordering* the fitness function is evaluated as in step 3, on the new offspring. The population is then rearranged by eliminating the chromosomes with the worst fitness values.

(8) *Loop statement* the best fitness value is compared to the threshold value and if the condition is matched the algorithm stops, while otherwise the entire process is repeated from step 3.

## Antenna design

**Figure 2:**
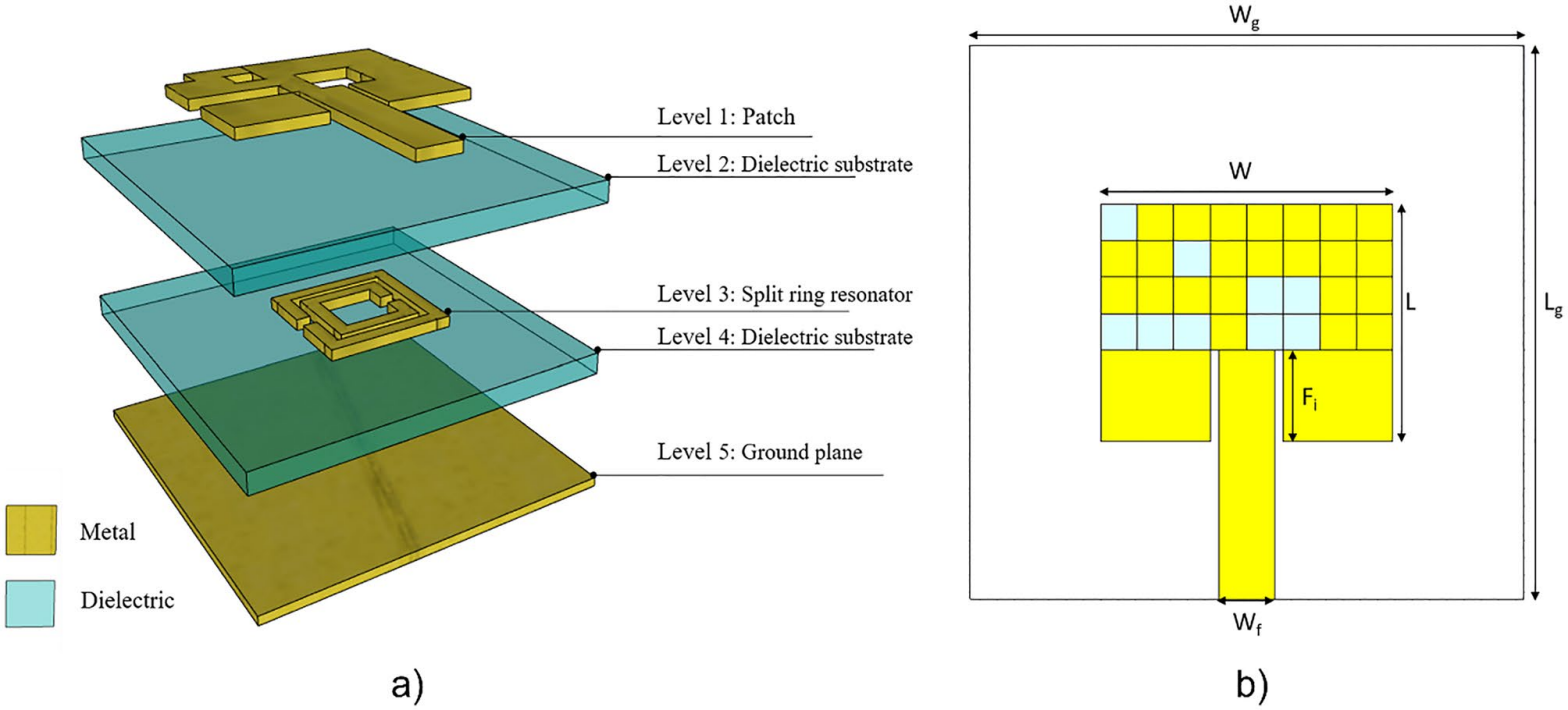
Sketches of the proposed antenna: (**a**) Exploded representation of the multilayer antenna, (**b**) top-view of the evolved patch antenna. Software used: Rhino 6, https://www.rhino3d.com/it/.

Figure 2a reports the structure of the evolved antenna.

The antenna is composed of 5 layers: from the top, the patch radiating element (level 1) which is on a dielectric substrate (level 2), then the metamaterial split ring resonator (level 3) placed on a second dielectric substrate (level 4) and the ground plane (level 5).

The dielectric substrates, made of PEN, have a thickness of 125 µm, a dielectric constant of 2.9 and a loss tangent equal to 0.005. PEN presents very good properties as a substrate for flexible electronics: it is a transparent and low-cost material. It is also characterized by a very high heat shrinkage coefficient and good resistance to acids and basis^28,29^.

### Evolved patch antenna miniaturization

The miniaturization is carried out starting from a classical patch antenna operating at 6 GHz with a bandwidth of 50 MHz and a gain equal to 5.8 dBi. The classical patch antenna can be realized by using the algorithm detailed in Ref.^30^. However, it is not possible to control and engineer the bandwidth or the gain of the device but only the geometrical parameters. Conversely, our optimization problem can be treated using a binary coding scheme, hence, the genetic algorithm is the most straightforward to be implemented. The geometrical parameters of the antenna (Fig. 2b) used in the simulation are listed in Table 1.

**Table 1:** Geometrical parameter of the considered patch antenna.

Parameter	Value (mm)
W	16.88
L	13.77
F_i_	5.26
Wf	3.19
W_g_	32
L_g_	32

The simulations have been performed considering the substrate as a loss-free dielectric layer having thickness equal to 125 µm.

The genetic algorithm implemented to miniaturize the antenna consists of the main steps described below.

(1) *Encoding* the encoding scheme used consists of subdividing the patch antenna into 32 pixels. A single pixel is treated as a gene and is encoded with a binary value, 1 if it is a metal pixel (yellow), 0 if it made of air (blue), as shown in Fig. 1b. Each pixel has a square form and an edge length of 2 µm.

(2) *Initialization* the starting elements are generated randomly. The population size is strictly linked to the nature of the problem and its degrees of freedom. By considering a huge population, it could be possible to obtain better results but at the cost of higher computational times; on the contrary, by using a small size for the population the execution speeds up but the optimizer may get stacked in local minima. To minimize the convergence time avoiding the insurgence of partial solutions, the possible elements of the population can be restricted to delete pointless individuals. In this case, since the radiation efficiency is directly linked to the size of the radiating area, each element of the population must be made of metal in a percentage greater than 60%.

(3) *Evaluation of the population* the value of the cost function ($\mathrm{C}\left({f}_{R}\right)$) of each antenna is evaluated by calculating the resonance f_R_ and the corresponding impedance value Z(f_R_), by means of a finite difference time domain (FDTD) solver using the Eq. (1):

1 $${\text{C}}\left( {f_{R} } \right) = \left\{ {\begin{array}{ll} {\left| {50 - Re\left\{ {Z(f_{R} )} \right\}} \right|e^{{\frac{{ - (f_{c} - f_{R} )}}{{f_{c} }}}} } & {f_{c} - \delta < f < f_{c} + \delta } \\ {1000} & {otherwhise} \\ \end{array} } \right.$$

where f_c_ is the objective frequency equal to 4 GHz, δ represents the tolerance to the resonant frequency and Z(f_R_) is the value of the impedance assumed by the patch at its resonance. The exponential term assumes lower values when the resonance f_R_ is near to the objective frequency. In addition, this function takes into account the impedance of the resonant peaks in order to discard antennas having a value of the real part of the impedance far from 50 Ω.

(4) *Chromosomes rank and selection process* the elements of the population are then grouped two by two using a roulette selection process. The probability associated with the selection of the single element π_i_ follows a Boltzmann distribution, hence is evaluated by means of Eq. (2):

2 $${\pi }_{i}={e}^{-\beta {c}_{i}}$$

where c_i_ is the value of the fitness function of the ith element of the population and β is a normalization factor equal to $\upbeta =\frac{1}{\mathrm{N}}\sum_{\mathrm{i}=1}^{\mathrm{N}}{\mathrm{c}}_{\mathrm{i}}$.

Elements characterized by a lower cost function, hence nearer to the final solution, result more attractive than the others; however, it is worth stressing that by using this selection process the probability of selecting the worse elements is not deprecated at all.

(5) *Crossover* two offsprings are then generated from each group by a single point cross-over.

(6) *Mutation* occasionally, a mutation event has occurred with a probability equal to 1%. The mutation corresponds to a variation of a single gene of an element of the population.

(7) *Reordering* the cost function is evaluated for each element of the new population. The elements are then reordered in an ascendent order with respect to their costs. The latest elements are discarded, and the best value is compared with the threshold value of the algorithm to decide on the convergence of the solution.

(8) *Loop statement* the process is repeated starting from step 3 until the condition on the threshold is reached.

In our implementation, the algorithm converged after 12 iterations giving as result an evolved patch antenna with a footprint of 16.88 × 13.76 mm^2^ resonating at around 3.89 GHz with an S_11_ dip of − 24.47 dB and a bandwidth of about 14 MHz.

The realized gain at the resonant frequency is equal to -0.647 dBi. The reduction of the dimensions of the antenna and, more important, of the ground plane always leads to worse radiation properties; to improve the antenna radiation pattern a mu-negative metamaterial composed of a square split ring resonator has been added between the top and the ground plane^31^.

## Split ring resonator

**Figure 3:**
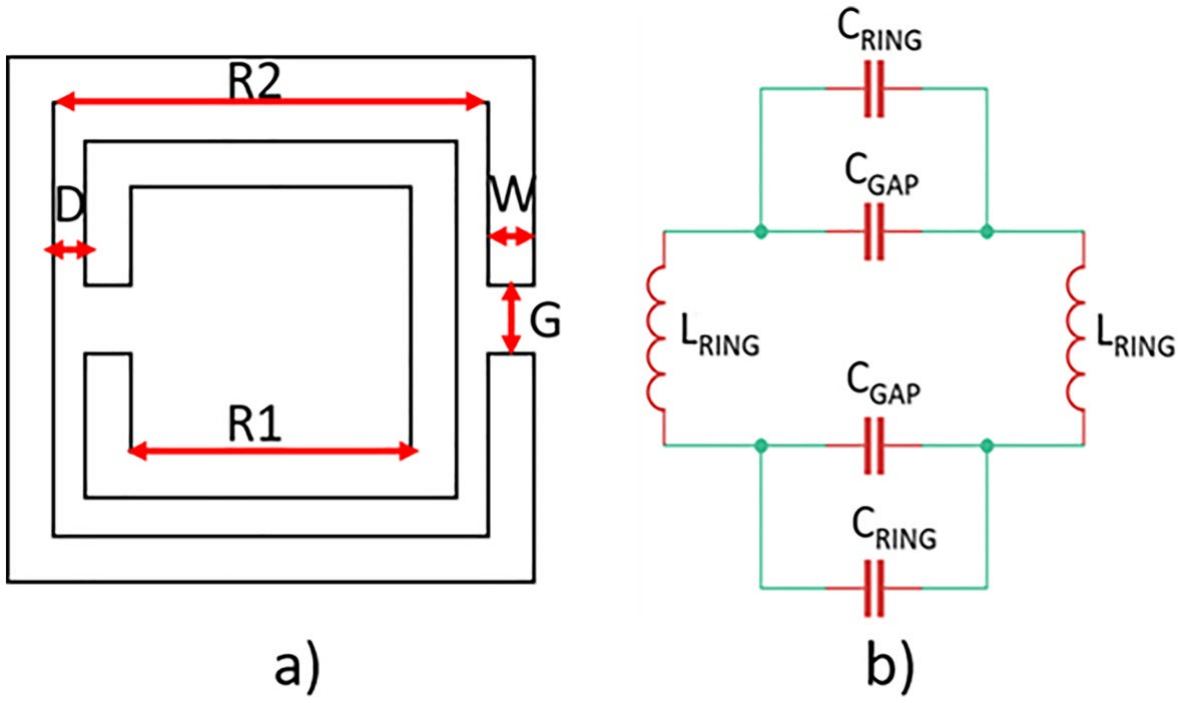
(**a**) Geometry and (**b**) equivalent circuit, of the SRR.

The square split ring resonator is composed of two concentric etched split rings separated by a gap and having their apertures placed in the opposite direction (Fig. 3a).

The SRR behaves like an LC circuit as shown in Fig. 3b^32^. The value of the inductance ${L}_{RING}$ of each ring can be expressed as in Eq. (3):

3 $${L}_{RING}=\frac{{\upmu }_{0}D}{W}[R2+R1]$$

D represents the gap between two rings, W is the ring thickness, R2 and R1 are the outer and inner radius of the rings, respectively.

As regards the capacitance, ${C}_{RING}$ represents the capacitance of the single ring while ${C}_{GAP}$ the capacitance between the rings, respectively. ${C}_{RING}$ can be defined as in the following expression, Eq. (4)

4 $${C}_{RING}=\frac{{\varepsilon }_{0}{\varepsilon }_{r}tW}{G}$$

where *t* is the thickness of the ring and G the aperture of the ring.

Finally, ${C}_{GAP}$ can be expressed as in Eq. (5):

5 $${C}_{GAP}=\frac{{A\varepsilon }_{0}{\varepsilon }_{r}W(2R2+2R1-G)}{2D}$$

A is an equilibrium constant.

By considering the geometrical parameters of the SRR, the resonant frequency ${f}_{0}$ can be evaluated as in Eq. (6):

6 $${f}_{0}=\frac{1}{2\pi \sqrt{{L}_{RING}({C}_{RING}+{C}_{GAP})}}\approx \frac{1}{2\pi \sqrt{{L}_{RING}{C}_{RING}}}$$

In this work, the geometry of the square split ring resonator has been properly designed to increase the gain at the working frequency of the antenna.

The geometrical dimensions of the square SRR are listed in Table 2.

**Table 2:** Geometrical parameters of the square SRR.

Parameter	Value (mm)
R1	4.5
R2	3
W	1
D	0.5

After this procedure, the entire structure is composed of 5 layers as shown in Fig. 2a.

**Figure 4:**
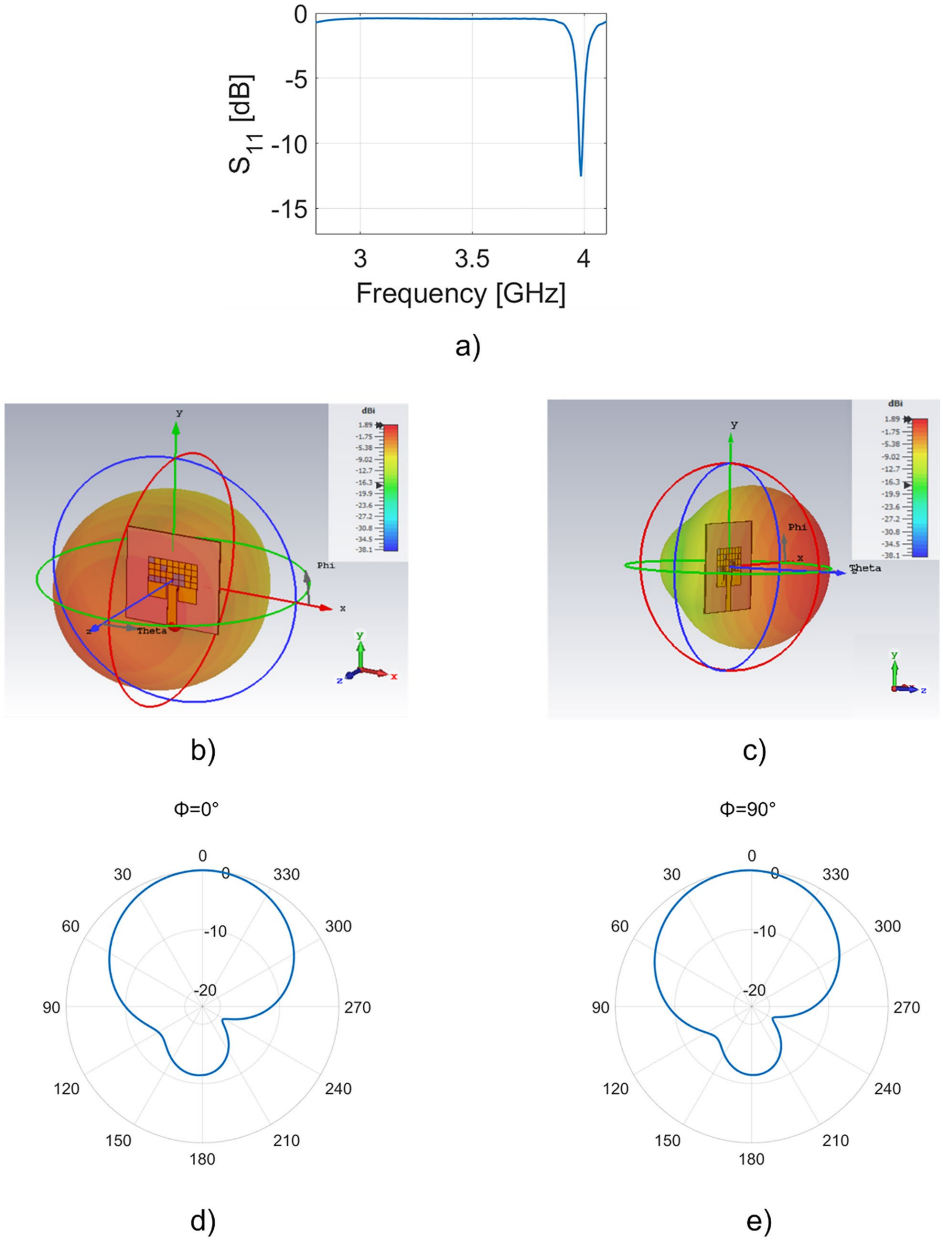
(**a**) Simulated S_11_ parameter of the multilayer antenna, (**b**) front perspective and (**c**) side perspective of 3D radiation patterns at 3.985 GHz; simulated polar plot for the plane (**d**) ϕ = 0° and (**e**) ϕ = 90°. Software used: figure a Matlab R2020a; (**b**–**d**) CST Microwave Studio 2021. https://it.mathworks.com/, https://www.3ds.com/products-services/simulia/products/cst-studio-suite/.

The results of the simulations are shown in Fig. 4.

In Fig. 4a the trend of S_11_ parameter is shown; there is a dip of − 14.2 dB at 3.984 GHz with a bandwidth of about 15 MHz. The narrow-band could be increased by redesigning the cost-function of our algorithm or considering a circular patch^33^. In Fig. 4b,c two different perspective of the 3D realized gain of the patch antenna at the resonant frequency are reported. The maximum value is equal to 1.89 dBi.

In Fig. 4d,e the radiation pattern in the E-plane and H-plane are reported, respectively. In particular, for the E-plane ($\phi =0^\circ$) the main lobe magnitude is equal to 1.89 dBi and the main lobe direction is equal to 3.0° with an angular width (3 dB) equals to 93.2°. Whereas the H-plane ($\phi =90^\circ$) the main lobe magnitude is equal to 1.88 dBi and the main lobe direction is equal to 0° with an angular width (3 dB) equals to 92°.

It is worth stressing that with the insertion of the dielectric layer with an SRR, it is possible to observe an increase of the gain from –0.647 dBi to 1.89 dBi without modifying the footprint of the patch antenna. The directivity is equal to 6.48 dBi at the resonant frequency.

Table 3 shows a comparison between the 6 GHz starting patch antenna, the 4 GHz evolved one and the 4 GHz classical patch antenna.

**Table 3:** Comparison between a classical and the evolved patch antenna.

	Classical patch antenna	Evolved patch antenna
Working frequency	6 GHz	3.96 GHz
Electrical length	$0.34\lambda \times 0.28\lambda$	$0.22\lambda \times 0.18\lambda$
Bandwidth	50 MHz	15 MHz
Gain	5.8 dBi	1.89 dBi
Physical dimensions	$17 \times 14\,\text{mm}^{2}$	$17 \times 14\,\text{mm}^{2}$

The Genetic Algorithm allows to retain the starting patch antenna footprint (17 × 14 mm^2^) at a lower working frequency of 3.96 GHz. In comparison with a classical patch antenna at the same frequency (whose physical dimension are 27 × 22 mm^2^), we got a reduction in size of 60%. Besides, the evolved patch antenna presents a reduction in terms of gain and bandwidth, in line with the behaviour of miniaturized and electrically small antennas^34^.

## Fabrication

The designed antenna has been fabricated by means of NanoDimesion’s DragonFly LDM™ 3D printer.

**Figure 5:**
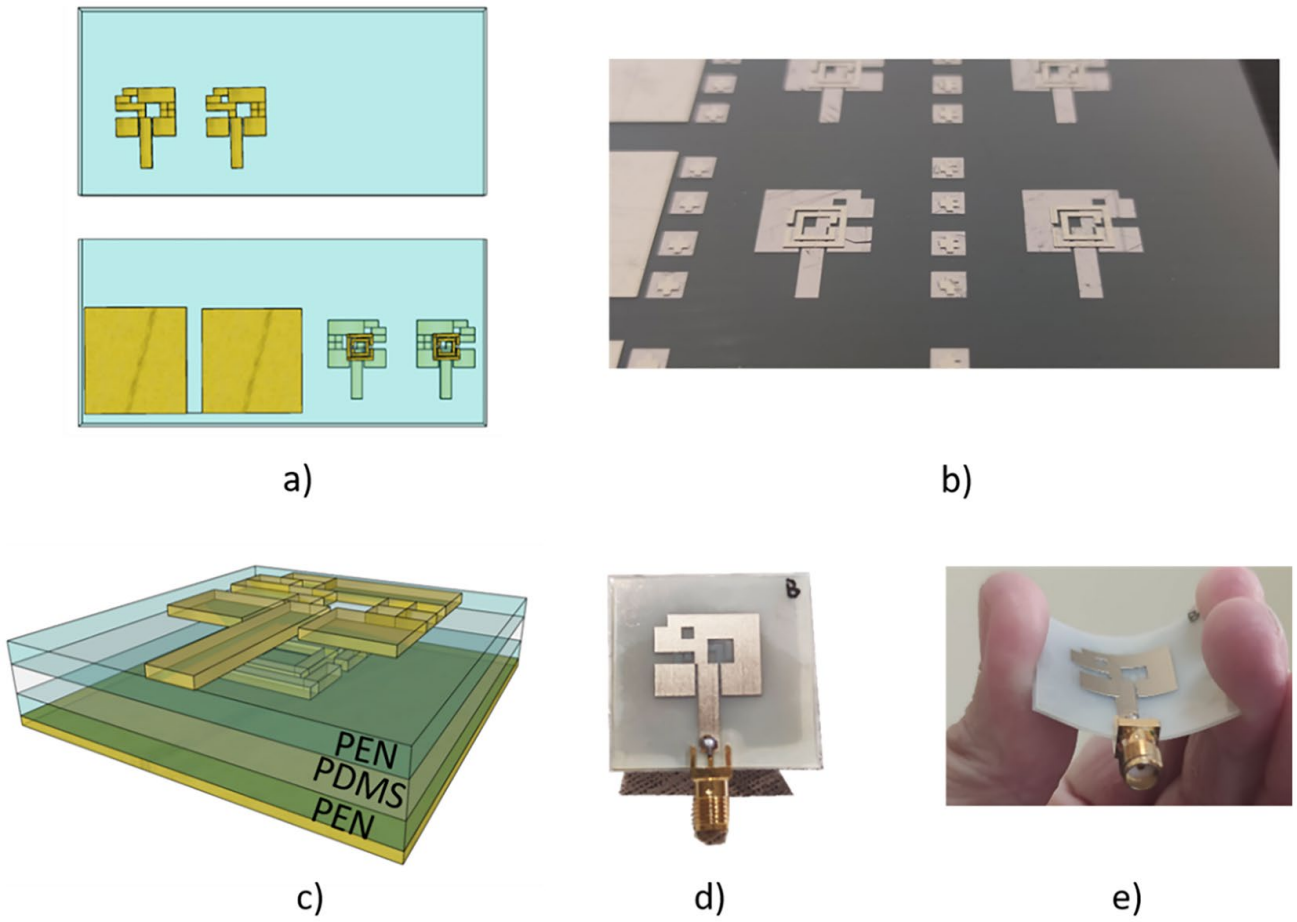
(**a**) Steps of the 3D printing process, (**b**) result of the alignment between patch and SRR and printed ground planes, (**c)** representation of the multilayer antenna with the interlayer of PDMS, (**d**) flat and (**e**) bent fabricated prototype with SMA connector. Software used: Rhino 6.

During the fabrication process, a material jetting of conductive ink through hundreds of nozzles has been performed. In particular, the metallic parts are made of AgCite, which is an Ag nanoparticles-based ink. All the process has been realized at a temperature of 140 °C necessary for the ink curing and sintering. The starting point of the print process is a complex 2D schematic of the device that is imported as a Gerber file and then converted into a layer-by-layer print instruction for multi-material 3D printer. Figure 5 exploits the fabrications steps.

More in detail, at the first stage the patch has been printed and then, a backside automated alignment follows for SRR printing on the bottom side. In parallel, the ground plane is printed near to the self-aligned SRR (Fig. 5a). The radiating elements have a thickness equal to 35 µm; as a technological constraint, the metal thickness should be at least equal to 17 µm to guarantee perfect electrical conductivity. In Fig. 5b, the alignment step realized on PEN substrate is reported. The two dielectric layers are cut by means of a laser cutter (Universal Laser System vls2.30) and then are joined with an adhesive interlayer of around 40 µm of Polydimethylsiloxane (PDMS), as reported in Fig. 5c. It is worth pointing out that the choice of this elastomer guarantees the flexibility of the antenna. Finally, Fig. 5d,e show the fabricated prototype.

## Characterization

The antenna has been characterized in terms of the scattering parameter S_11_ and the radiation pattern.

The return loss has been measured by means of a Vector Network Analyzer (VNA, Anritsu MS46122B).

**Figure 6:**
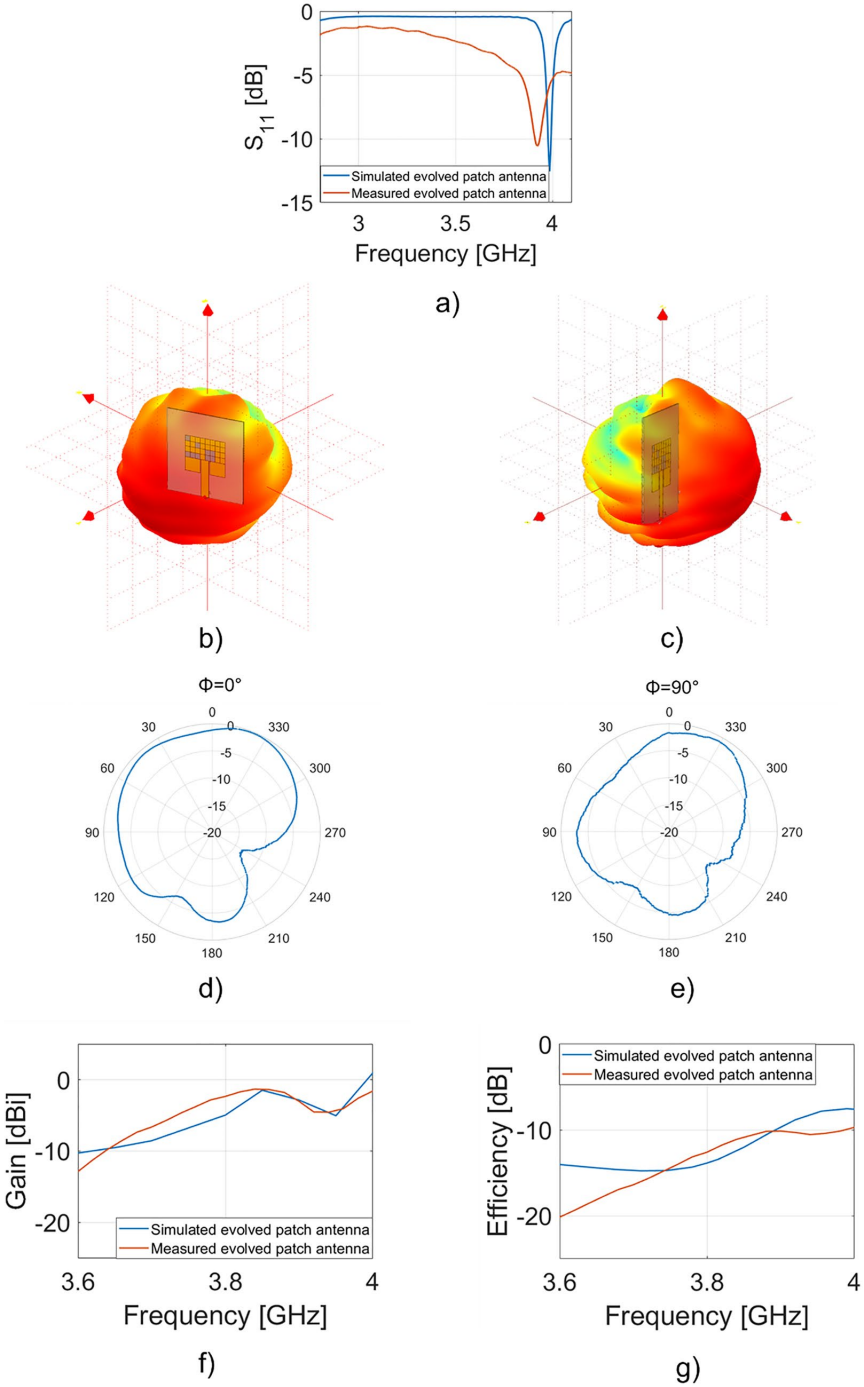
(**a**) Simulated (blue curve) and measured (red curve) S_11_ parameter of the multilayer antenna, measured (**b**) front perspective and (**c**) side perspective 3D radiation patterns; polar plot at (**d**) ϕ = 0° and (**e**) ϕ = 90° at 4 GHz; simulated (blue curve) and measured (red curve) (**f**) gain and (**g**) efficiency of the evolved patch antenna. Software used: proprietary software by Starlab from Satimo 18 GHz anechoic chamber, https://www.mvg-world.com/en/products/antenna-measurement/multi-probe-systems/starlab.

Figure 6a shows a comparison between the trends of the simulated (blue curve) and the measured (red curve) scattering parameter S_11_.

In particular, it is possible to see a dip of − 14.2 dB at 3.984 GHz and a dip of -13.41 dB at 3.962 GHz for the simulated and the measured evolved patch antenna, respectively. There is a shift of few tens of MHz due to the introduction of a SMA connector which introduces a static capacitance, therefore increasing the electrical length of the device^35^. Both curves present a dip around 4 GHz, the desired operating frequency, where the realized gain is equal to -0.8 dBi for the simulated antenna and -1.5 dBi for the fabricated one, when the losses are taken into account. Figure 6b,c show the measured 3D radiation patterns that are in agreement with the simulated ones (Fig. 4b,c). 2D polar plots and 3D radiation diagram were measured by means of the combination of an anechoic chamber (StarLab from Satimo) and a home-made setup. In Fig. 6d,e the measured radiation patterns for E-plane and H-plane are reported. For the planes ϕ = 0° and ϕ = 90°, there is a very good agreement between simulations (Fig. 4d,e) and measurements (Fig. 6d,e). Figure 6f shows the gain around the resonant frequency equal to 3.9 GHz: as it can be inferred by the plot, there is a good agreement between the numerical and experimental results. As regards the efficiency shown in Fig. 6g, it is possible to note a higher value of the simulated one with respect to the measured one at the resonant frequency. It is worth stressing that this mismatch is due to multiple factors such as: the soldering of the connector, the presence of solvent mixed with the conductive part used in the 3D printing process and finally, the losses of the substrate at the resonant frequency that has been extrapolated from the lower frequencies value tabulated in the PEN datasheet (1 kHz-1 GHz) [Teonex Q51].

## Conclusion

A multi-layer 3D-printed metamaterial-based evolved patch antenna has been presented. The genetic algorithm has proven to be a powerful method to optimize antenna miniaturization and the radiation properties have been improved with the insertion of the split-ring resonator. The fabrication protocol turns out to be very fast and capable of realizing antennas with a precise alignment step between the layers. The measurements of the S_11_ parameter and the radiation patterns are in agreement with the simulations. The main resonance of the fabricated prototype is in the sub-6 GHz of 5G spectrum, showing a dip of S_11_ parameter of − 27.41 dB at 3.962 GHz, with a bandwidth of 14.7 MHz. In case directivity and gain need to be enhanced, an array configuration of this patch can be implemented*.* We believe that the proposed approach could be a valid choice for the realization of compact, flexible and wearable IoT devices.

## Data Availability

All the data supporting the results of this study can be found within the article or upon request from the corresponding authors.
